# Supplementary figures and images for: Mitochondrial genome in sporadic breast cancer: A case control study and a proteomic analysis in a Sinhalese cohort from Sri Lanka
Source: PLoS One. 2023 Feb 9;18(2):e0281620. doi: 10.1371/journal.pone.0281620 (PMC9910733; doi:10.1371/journal.pone.0281620)

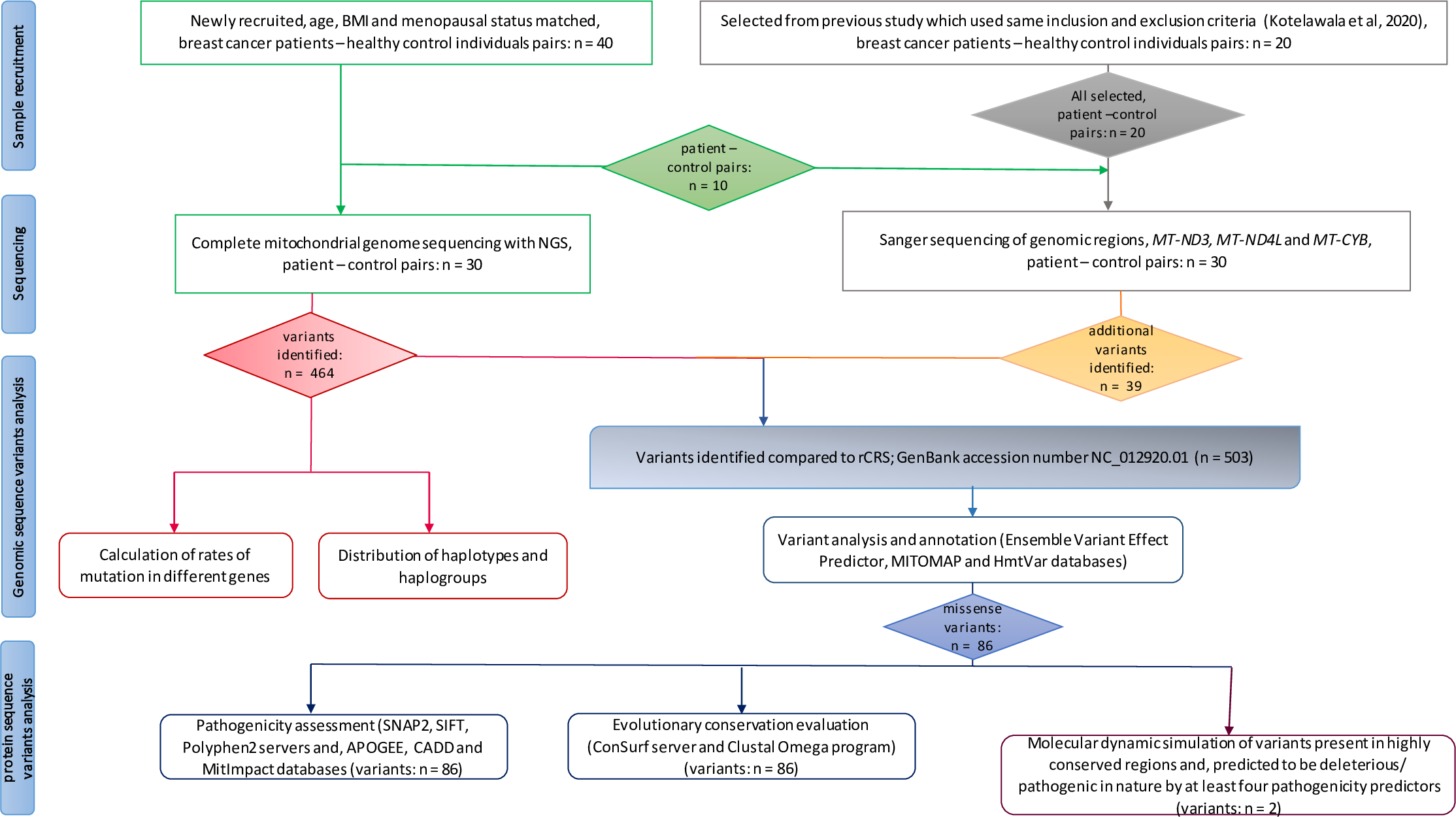

Supplement: S1 Fig — (TIF) [file pone.0281620.s001.tif]

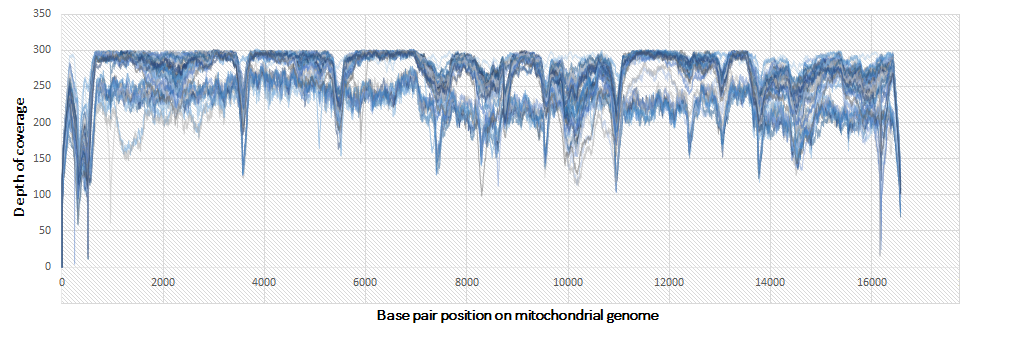

Supplement: S2 Fig — (TIF) [file pone.0281620.s002.tif]
