# Supplementary material for: Mitochondrial genome in sporadic breast cancer: A case control study and a proteomic analysis in a Sinhalese cohort from Sri Lanka
Source: PLoS One. 2023 Feb 9;18(2):e0281620. doi: 10.1371/journal.pone.0281620 (PMC9910733; doi:10.1371/journal.pone.0281620)
Supplement: S1 Table — (DOCX) [file pone.0281620.s003.docx]

**Supplementary Table 1. Body size of sporadic breast cancer patients and matched controls (N=60 pairs)**

| Body size (BMI range)^a^ | Patients | Controls |
| --- | --- | --- |
| Underweight (< 18.5) | 3 (5.00%) | 3 (5.00%) |
| Normal (18.5 - 22.9) | 12 (20.00%) | 13 (21.66%)^b^ |
| Overweight ( 23 – 24.9) | 12 (20.00%) | 11(18.33%)^b^ |
| Obese (>25) | 33 (55.00) | 33 (55.00%) ^b^ |

^a^As per Asia Pacific cut off values for Body Mass Index (BMI)

<http://www.wpro.who.int/nutrition/documents/docs/Redefiningobesity.pdf>;

^b^During stratification by body size control of one patient in the overweight group fell into the normal, as matching was done within one unit of BMI. Similarly, a control of one patient in the overweight group fell into the obese group, and a control of a patient in the obese group fell into the overweight group. When body size groups were compared the individual numbers shown above were used.
