# Supplementary material for: Mitochondrial genome in sporadic breast cancer: A case control study and a proteomic analysis in a Sinhalese cohort from Sri Lanka
Source: PLoS One. 2023 Feb 9;18(2):e0281620. doi: 10.1371/journal.pone.0281620 (PMC9910733; doi:10.1371/journal.pone.0281620)
Supplement: S2 Table — (DOCX) [file pone.0281620.s004.docx]

**Supplementary Table 2: Histological type of cancer in 60 sporadic breast cancer patients**

| Histological type | Number | Percentage |
| --- | --- | --- |
| Invasive ductal carcinoma [ie: not otherwise specified (NOS) and carcinoma of no special type (NST) | 45 | 75% |
| Invasive lobular carcinoma | 6 | 10.00% |
| Ductal carcinoma in situ | 2 | 3.33% |
| Invasive ductal (NST) and mucinous carcinoma | 2 | 3.33% |
| Mixed and other types | 5 | 8.3% |
