# Supplementary material for: Mitochondrial genome in sporadic breast cancer: A case control study and a proteomic analysis in a Sinhalese cohort from Sri Lanka
Source: PLoS One. 2023 Feb 9;18(2):e0281620. doi: 10.1371/journal.pone.0281620 (PMC9910733; doi:10.1371/journal.pone.0281620)
Supplement: S3 Table — (DOCX) [file pone.0281620.s005.docx]

**Supplementary Table 3. Variants identified at >5% within either population (patients: N=30; controls: N= 30) in the coding region of the mt genome by next generation sequencing excluding genomic regions *MT-ND3, MT-ND6, MT-ND4L*, *MT-CYB, MT-TT***

| Map locus of the human MT-genome | Variant | Nature  of the variant | Codon change | Amino  acid  change | Patients  n (%) | Controls  n (%) |
| --- | --- | --- | --- | --- | --- | --- |
| *MT-RNR1* | A750G | - | - | - | 30 (100.00) | 30 (100.00) |
| *MT-RNR1* | 955insC | - | - | - | - | 2 (6.66) |
| *MT-RNR1* | 961delT | - | - | - | - | 2 (6.66) |
| *MT-RNR1* | T961C | - | - | - | - | 2 (6.66) |
| *MT-RNR1* | T980C | - | - | - | 2 (6.66) | 2 (6.66) |
| *MT-RNR1* | A1438G | - | - | - | 30 (100.00) | 30 (100.00) |
| *MT-TV* | G1664A | - | - | - | 4 (13.33) | 4 (13.33) |
| *MT - RNR2* | A1811G | - | - | - | 5 (16.66) | 9 (30.00) |
| *MT - RNR2* | G1888A | - | - | - | 1 (3.33) | 4 (13.33) |
| *MT-RNR2* | C2218T | - | - | - | 2 (6.66) | - |
| *MT - RNR2* | A2706G | - | - | - | 28 (93.32) | 27 (90.00) |
| *MT-RNR2* | G2831T | - | - | - | 2 (6.66) | - |
| *MT – ND1* | T3336C | syn | atT/atC | I | 4 (13.33) | 4 (13.33) |
| ***MT-ND1*** | **A3434G** | mis | tAc/tGc | Y/C | 2 (6.66) | - |
| *MT-ND1* | C3486T | syn | ccC/ccT | P | - | 2 (6.66) |
| *MT-ND1* | A3537G | syn | ttA/ttG | L | - | 3 (10.00) |
| *MT – ND1* | C3741T | syn | acC/acT | T | 2 (6.66) | 2 (6.66) |
| *MT-ND1* | A4188G | - | - | - | - | 2 (6.66) |
| *MT-TQ* | C4394T | - | - | - | 3 (10.00) | - |
| ***MT-ND2*** | **G4491A** | mis | Gtc/Atc | V/I | 4 (13.33) | - |
| *MT-ND2* | G4991A | syn | caG/caA | Q | 2 (6.66) | - |
| ***MT-ND2*** | **T4561C** | mis | gTa/gCa | V/A | 2 (6.66) | 1 (3.33) |
| ***MT-ND2*** | **C4640A** | mis | atC/atA | I/M | - | 2 (6.66) |
| *MT- ND2* | A4769G | syn | atA/atG | M | 30 (100.00) | 30 (100.00) |
| *MT- ND2* | A4916G | syn | ctA/ctG | L | 4 (13.33) | 4 (13.33) |
| *MT-ND2* | T5082C | syn | Tta/Cta | L | - | 3 (10.00) |
| ***MT-ND2*** | **A5186T** | mis | tgA/tgT | W/C | 1 (3.33) | 2 (6.66) |
| ***MT-ND2*** | **A5301G** | mis | Atc/Gtc | I/V |  | 3 (10.00) |
| *MT -ND2* | C5360T | syn | atC/atT | I | 2 (6.66) | 2 (6.66) |
| *MT- ND2* | T5426C | syn | caT/caC | H | 2 (6.66) | 4 (13.33) |
| *MT -ND2* | A5432G | syn | aaA/aaG | K | 2 (6.66) | 3 (10.00) |
| MT-TW | A5558G | - | - | - | - | 3 (10.00) |
| *MT-TC*/MT-OLR | C5790A | - | - | - | 2 (6.66) | 1 (3.33) |
| *MT-CO 1* | G6026A | syn | ctG/ctA | L | 2 (6.66) |  |
| ***MT-CO I*** | **A6040G** | mis | aAc/aGc | N/S | 4 (13.33) | 4 (13.33) |
| *MT-CO I* | T6620C | syn | ggT/ggC | G | 3 (10.00) | 1 (3.33) |
| *MT-CO I* | C7028T | syn | gcC/gcT | A | 28 (93.32) | 29 (96.66) |
| *MT-TD* | T7581C | - | - | - | 2 (6.66) | - |
| *MT-CO 2* | C7777T | syn | gtC/gtT | V | - | 2 (6.66) |
| ***MT-CO 2*** | **G7859A** | mis | Gat/Aat | D/N | 2 (6.66) | - |
| *MT-CO 2* | T8023C | syn | atT/atC | I | 2 (6.66) | 1 (3.33) |
| *MT-CO 2* | C8137T | syn | ttC/ttT | F | 2 (6.66) | 3 (10.00) |
| *MT-CO 2* | G8251A | syn | ggG/ggA | G | 5 (16.66) | 4 (13.33) |
| MT-NC7 | 8270  insACCCCCTCT | - | - | - | 2 (6.66) | 1 (3.33) |
| ***MT-ATP8/ MT-ATP6*** | **G8572A*** | mis | Ggc/Agc | G/S | - | 2 (6.66) |
| *MT-ATP6* | C8676T | syn | atC/atT | I | 2 (6.66) | 1 (3.33) |
| ***MT-ATP6*** | **C8684T** | mis | aCc/aTc | T/I | 2 (6.66) | 2 (6.66) |
| ***MT-ATP6*** | **A8701G** | mis | Acc/Gcc | T/A | 17 (56.66) | 18 (60.00) |
| *MT-ATP6* | A8739G | syn | atA/atG | M | 4 (13.33) | 4 (13.33) |
| ***MT-ATP6*** | **A8860G** | mis | Aca/Gca | T/A | 30 (100.00) | 29 (96.66) |
| *MT-ATP6* | C9094T | mis | Ctt/Ttt | L/F | 1 (3.33) | 2 (6.66) |
| *MT-CO 3* | G9329A | syn | acG/acA | T | - | 2 (6.66) |
| *MT-CO 3* | T9540C | syn | Tta/Cta | L | 17 (56.66) | 18 (60.00) |
| *MT-CO 3* | A9614G | syn | gtA/gtG | V | 1 (3.33) | 2 (6.66) |
| *MT-CO 3* | T9656C | syn | agT/agC | S | - | 2 (6.66) |
| *MT-CO 3* | C9767T | syn | acC/acT | T | 2 (6.66) | 1 (3.33) |
| *MT-CO 3* | G9947A | syn | gtG/gtA | V | - | 2 (6.66) |
| ***MT-CO 3*** | **G9966A** | mis | Gtc/Atc | V/I | - | 2 (6.66) |
| *MT-ND4* | T10873C | syn | ccT/ccC | P | 17 (56.66) | 18 (60.00) |
| ***MT-ND4*** | **T11253C** | mis | aTt/aCt | I/T | 1 (3.33) | 2 (6.66) |
| *MT-ND4* | A11467G | syn | ttA/ttG | L | 7 (23.33) | 9 (35.00) |
| *MT-ND4* | G11719A | syn | ggG/ggA | G | 28 (93.32) | 29 (96.66) |
| *MT-ND4* | G12007A | syn | tgG/tgA | W | 12 (40.00) | 10 (33.33) |
| *MT-ND4* | C12106T | syn | ctC/ctT | L | 1 (3.33) | 2 (6.66) |
| *MT-TS2* | A12234G | - | - | - | 1 (3.33) | 2 (6.66) |
| *MT-TL2* | A12308G | - | - | - | 7 (23.33) | 9 (35.00) |
| *MT-ND5* | G12372A | syn | ctG/ctA | L | 7 (23.33) | 9 (35.00) |
| *MT-ND5* | G12561A | syn | caG/caA | Q | 2 (6.66) | 3 (10.00) |
| *MT-ND5* | C12705T | syn | atC/atT | I | 18 (60.00) | 18 (60.00) |
| *MT-ND5* | T12879C | syn | ggT/ggC | G | 4 (13.33) | - |
| *MT-ND5* | T13020C | syn | ggT/ggC | G | - | 2 (6.66) |
| *MT-ND5* | A13104G | syn | ggA/ggG | G | 2 (6.66) | - |
| *MT-ND5* | G13194A | syn | ctG/ctA | L | 1 (3.33) | 3 (10.00) |
| *MT-ND5* | T13500C | syn | ggT/ggC | G | 2 (6.66) | 2 (6.66) |
| ***MT-ND5*** | **A13651G** | mis | Acc/Gcc | T/A | 4 (13.33) | 4 (13.33) |
| *MT-ND5* | T13656C | syn | ctT/ctC | L | - | 2 (6.66) |
| *MT-ND5* | T13743C | syn | acT/acC | T | - | 2 (6.66) |
| *MT-ND5* | A13803T | syn | acA/acT | T | 2 (6.66) | - |
| ***MT-ND5*** | **A13966G** | mis | Acg/Gcg | T/A | - | 2 (6.66) |
| *MT-ND5* | A14070G | syn | tcA/tcG | S | 2 (6.66) | - |
| ***MT-ND5*** | **A14128G** | mis | Acc/Gcc | T/A | - | 3 (10.00) |
| *MT-ND5* | A14139G | syn | ctA/ctG | L | - | 2 (6.66) |
| MT-NC10 | A15954C | - | - | - | 2 (6.66) | - |

ATP: Adenosine triphosphate synthase F0 subunit, CO: Cytochrome c oxidase subunit, NC: Non-coding nucleotides, ND: NADH (nicotinamide adenine dinucleotide + hydrogen) dehydrogenase subunit, RNR1: 12S ribosomal RNA (ribonucleic acid), RNR2: 16S ribosomal RNA, TC/OLR: tRNA cysteine/ L-strand origin, TL: tRNA leucine, TQ: tRNA glutamine, TS: tRNA serine, TV: tRNA valine, TW: tRNA tryptophan

mis: missense, syn: synonymous

*G8572A mutation serves a dual function; in *MT*-*ATP8* gene as a stop-retained variant and in *MT*-*ATP6* gene as a missense mutation
