# Supplementary material for: Mitochondrial genome in sporadic breast cancer: A case control study and a proteomic analysis in a Sinhalese cohort from Sri Lanka
Source: PLoS One. 2023 Feb 9;18(2):e0281620. doi: 10.1371/journal.pone.0281620 (PMC9910733; doi:10.1371/journal.pone.0281620)
Supplement: S4 Table — (DOCX) [file pone.0281620.s006.docx]

**Supplementary Table 4. Variants identified at >5% within either population (patients: N=60; controls: N= 60) in the genomic regions *MT-ND3, MT-ND6, MT-ND4L*, *MT-CYB, MT-TT* by next generation sequencing and Sanger sequencing**

| Map locus of the human MT-genome | Variant | Nature of the variant | Codon change | Amino acid change | Patients  n (%) | Controls  n (%) |
| --- | --- | --- | --- | --- | --- | --- |
| ***MT-ND3*** | **A10398G** | **missense** | Acc/Gcc | T/A | 35 (58.33) | 35 (58.33) |
| *MT-ND3* | C10400T | synonymous | acC/acT | T | 35 (58.33) | 35 (58.33) |
| *MT-ND4L* | C10670T | synonymous | gcC/gcT | A | 6 (10.00) | 6 (10.00) |
| *MT-ND6* | T14290C | synonymous | gaA/gaG | E | 7 (11.66) | 5 (8.33) |
| ***MT-CYB*** | **C14766T** | **missense** | aCt/aTt | T/I | 57 (95) | 59 (98.33) |
| *MT-CYB* | T114783C | synonymous | Tta/Cta | L | 36 (60.00) | 35 (58.33) |
| *MT – CYB* | G14905A | synonymous | atG/atA | M | 2 (3.33) | 4 (6.66) |
| *MT-CYB* | G15043A | synonymous | ggG/ggA | G | 36 (60.00) | 35 (58.33) |
| *MT – CYB* | C15049T | synonymous | ggC/ggT | G | 1 (1.66) | 4 (6.66) |
| ***MT – CYB*** | **G15110A** | **missense** | Gca/Aca | A/T | 2 (3.33) | 4 (6.66) |
| *MT-CYB* | G15148A | synonymous | ccG/ccA | P | 4 (6.66) | 4 (6.66) |
| *MT-CYB* | G15301A | synonymous | ttG/ttA | L | 35 (58.33) | 35 (58.33) |
| ***MT – CYB*** | **G15314A** | **missense** | Gca/Aca | A/T | 3 (5.00) | 4 (6.66) |
| ***MT-CYB*** | **A15326G** | **missense** | Aca/Gca | T/A | 56 (93.33) | 59 (98.33) |
| ***MT-CYB*** | **G15431A** | **missense** | Gcc/Acc | A/T | 6 (10.00) | 5 (8.33) |
| ***MT – CYB*** | **G15884A** | **missense** | Gcc/Acc | A/T | 3 (5.00) | 4 (6.66) |
| *MT-TT* | A15924G | non coding exon | - | - | 14 (23.33) | 11 (18.33) |

CYB: Cytochrome b, ND: NADH (nicotinamide adenine dinucleotide + hydrogen) dehydrogenase subunit, TT: tRNA (ribonucleic acid) threonine
