# Supplementary material for: Mitochondrial genome in sporadic breast cancer: A case control study and a proteomic analysis in a Sinhalese cohort from Sri Lanka
Source: PLoS One. 2023 Feb 9;18(2):e0281620. doi: 10.1371/journal.pone.0281620 (PMC9910733; doi:10.1371/journal.pone.0281620)
Supplement: S5 Table — (DOCX) [file pone.0281620.s007.docx]

**Supplementary Table 5 – All the missense variations identified and the prediction of protein function and pathogenicity**

| Variant | Patients | Controls | Prediction | | | | Amino acid change | Codon change |  |  |
| --- | --- | --- | --- | --- | --- | --- | --- | --- | --- | --- |
|  | n (%) | n (%) | SIFT | POLY-PHEN2 | CADD | APOGEE |  |  | SNAP2 score | ConSurf score |
| *MT-ND1* of 30 matched pairs | | | | | | | | |  |  |
| G3316A | _ | 1 (3.33) | T (lc) | B | N | N | A/T | Gcc/Acc | 2 | 1 |
| T3394C | _ | 1 (3.33) | D (lc) | B | N | **P** | Y/H | Tat/Cat | 87 | 4 |
| A3397G | 1 (3.33) | _ | D (lc) | B | N | **P** | M/V | Ata/Gta | 57 | 7 |
| G3421A | _ | 1 (3.33) | T (lc) | B | N | N | V/I | Gtt/Att | -98 | 7 |
| A3434G | 2 (6.66) | _ | D (lc) | B | N | N | Y/C | tAc/tGc | -45 | 1 |
| C3533T | _ | 1 (3.33) | T (lc) | B | N | N | T/I | aCc/aTc | -70 | 3 |
| A3865G | _ | 1 (3.33) | T (lc) | B | N | N | I/V | Atc/Gtc | -84 | 6 |
| C4099T | _ | 1 (3.33) | T (lc) | B | **D** | N | L/F | Ctt/Ttt | -76 | 1 |
| *MT-ND2* of 30 matched pairs | | | | | | | | |  |  |
| G4491A | 4 (13.33) | _ | T | B | N | N | V/I | Gtc/Atc | -63 | 3 |
| T4561C | 2 (6.66) | 1 (3.33) | T | B | N | N | V/A | gTa/gCa | -67 | 7 |
| **A4638G** | _ | 1 (3.33) | **D** | B | N | N | I/V | Atc/Gtc | -9 | 8 |
| **C4640A** | _ | 2 (6.66) | **D** | **PSD** | **D** | **P** | I/M | atC/atA | 53 | 8 |
| C4654T | _ | 1 (3.33) | T | B | **D** | N | T/M | aCg/aTg | -51 | 7 |
| C4938A | 1 (3.33) | _ | T | B | N | N | L/I | Ctc/Atc | -69 | 4 |
| G4959A | 1 (3.33) | _ | T | B | N | N | A/T | Gca/Aca | -56 | 4 |
| A5097G | 1 (3.33) | _ | T | B | N | N | I/V | Atc/Gtc | -84 | 1 |
| **A5186T** | 1 (3.33) | 2 (6.66) | **D** | **PRD** | **D** | **P** | W/C | tgA/tgT | 14 | 1 |
| A5301G | _ | 3 (10.00) | T | B | N | N | I/V | Atc/Gtc | 28 | 1 |
| **A5319G** | _ | 1 (3.33) | **D** | B | **D** | N | T/A | Acc/Gcc | 1 | 5 |
| C5461T | _ | 1 (3.33) | T | B | N | N | A/V | gCc/gTc | 67 | 1 |
| **T5503C** | _ | 1 (3.33) | T | **PSD** | N | N | M/T | aTa/aCa | -75 | 2 |
| *MT-CO1* of 30 matched pairs | | | | | | | | |  |  |
| C5911T | 1 (3.33) | _ | T (lc) | B | N | **P** | A/V | gCc/gTc | -99 | 1 |
| A6040G | 4 (13.33) | 4 (13.33) | T (lc) | B | N | N | N/S | aAc/aGc | -94 | 1 |
| **G6249A** | _ | 1 (3.33) | D (lc) | B | **D** | N | A/T | Gct/Act | -57 | 3 |
| **G6267A** | _ | 1 (3.33) | D (lc) | B | **D** | **P** | A/T | Gca/Aca | -22 | 4 |
| A7149G | _ | 1 (3.33) | T (lc) | B | N | N | I/V | Atc/Gtc | -84 | 1 |
| T7270C | 1 (3.33) | _ | D (lc) | B | N | N | V/A | gTa/gCa | -9 | 1 |
| *MT-CO2* of 30 matched pairs | | | | | | | | |  |  |
| G7775A | _ | 1 (3.33) | T (lc) | B | N | N | V/I | Gtc/Atc | -87 | 4 |
| **G7859A** | 2 (6.66) | _ | T (lc) | B | N | **P** | D/N | Gat/Aat | -74 | 1 |
| *MT-ATP8* of 30 matched pairs | | | | | | | | |  |  |
| T8400C | _ | 1 (3.33) | T | B | N | N | M/T | aTa/aCa | 37 | 7 |
| A8456G | _ | 1 (3.33) | T | B | N | N | T/A | Aca/Gca | 17 | 3 |
| **A8502G** | 1 (3.33) | _ | T | **PSD** | N | **P** | N/S | aAt/aGt | -13 | 1 |
| *MT-ATP6* of 30 matched pairs | | | | | | | | |  |  |
| **G8572A** | _ | 2 (6.66) | **D** | **PSD** | **D** | N | G/S | Ggc/Agc | 78 | 2 |
| G8584A | 1 (3.33) | _ | T | B | N | N | A/T | Gca/Aca | -39 | 6 |
| T8618C | 1 (3.33) | 1 (3.33) | T | B | N | N | I/T | aTc/aCc | 33 | 2 |
| C8684T | 2 (6.66) | 2 (6.66) | T | B | N | N | T/I | aCc/aTc | 54 | 6 |
| A8701G | 17 (56.66) | 18 (60.00) | T | B | N | N | T/A | Acc/Gcc | 41 | 1 |
| **T8705C** | 1 (3.33) | _ | T | B | N | **P** | M/T | aTa/aCa | 65 | 7 |
| **A8812G** | _ | 1 (3.33) | T | **PRD** | **D** | N | T/A | Acc/Gcc | 40 | 9 |
| T8843C | 1 (3.33) | _ | T | B | N | N | I/T | aTc/aCc | -6 | 6 |
| A8860G | 30 (100.00) | 29 (96.66) | T | B | N | N | T/A | Aca/Gca | 66 | 7 |
| **A8962G** | 1 (3.33) | _ | **D** | **PRD** | **D** | N | T/A | Acc/Gcc | 56 | 8 |
| G9064A | 1 (3.33) | _ | T | B | N | N | A/T | Gca/Aca | -44 | 1 |
| **C9094T** | 1 (3.33) | 2 (6.66) | T | **PSD** | **D** | N | L/F | Ctt/Ttt | -58 | 1 |
| *MT-CO3* of 30 matched pairs | | | | | | | | |  | |
| **G9438A** | 1 (3.33) | _ | D (lc) | B | **D** | **P** | G/S | Ggc/Agc | 83 | 6 |
| A9468G | 1 (3.33) | 1 (3.33) | T (lc) | B | N | N | T/A | Acc/Gcc | -66 | 4 |
| A9852G | 1 (3.33) | 1 (3.33) | T (lc) | B | N | N | T/A | Act/Gct | -63 | 1 |
| G9966A | _ | 2 (6.66) | T (lc) | B | N | N | V/I | Gtc/Atc | 33 | 3 |
| *MT-ND3* of 60 matched pairs | | | | | | | | |  | |
| A10188G | _ | 1 (1.66) | N | B | N | N | M/V | Ata/Gta | 10 | 5 |
| G10365A | 2 (3.33) | 1 (1.66) | T (lc) | B | N | N | A/T | Gcc/Acc | -21 | 5 |
| A10398G | 35 (58.33) | 35 (58.33) | T (lc) | B | N | N | T/A | Acc/Gcc | -6 | 5 |
| *MT-ND4L* of 60 matched pairs | | | | | | | | |  | |
| T10707G | 1 (1.66) | _ | - | - | N | N | S/A | Tca/Gca | -33 | 7 |
| *MT-ND4* of 30 matched pairs | | | | | | | | |  | |
| **G11016A** | _ | 1 (3.33) | T (lc) | B | N | **P** | S/N | aGt/aAt | -99 | 1 |
| C11061T | _ | 1 (3.33) | T (lc) | B | N | N | S/F | tCc/tTc | -44 | 1 |
| C11151T | _ | 1 (3.33) | T (lc) | B | N | N | A/V | gCt/gTt | -89 | 5 |
| **T11253C** | 1 (3.33) | 2 (6.66) | T (lc) | B | N | **P** | I/T | aTt/aCt | 23 | 5 |
| T11255C | 1 (3.33) | _ | T (lc) | B | N | N | Y/H | Tac/Cac | 5 | 1 |
| **G11453A** | _ | 1 (3.33) | D (lc) | **PRD** | **D** | **P** | A/T | Gcc/Acc | 14 | 9 |
| **T11916A** | 1 (3.33) | _ | D (lc) | **PRD** | **D** | **P** | F/Y | tTc/tAc | -15 | 5 |
| G11963A | _ | 1 (3.33) | T (lc) | B | N | N | V/I | Gtc/Atc | -50 | 7 |
| *MT-ND5* of 30 matched pairs | | | | | | | | |  | |
| C12346T | 1 (3.33) | _ | T (lc) | U | N | N | H/Y | Cac/Tac | -78 | 2 |
| A12437G | 1 (3.33) | _ | T (lc) | **PRD** | N | N | H/R | cAt/cGt | -39 | 1 |
| A13105G | 1 (3.33) | _ | T (lc) | S | N | N | I/V | Atc/Gtc | -35 | 9 |
| A13651G | 4 (13.33) | 4 (13.33) | T (lc) | **PRD** | **D** | N | T/A | Acc/Gcc | -21 | 1 |
| **G13708A** | 1 (3.33) | 1 (3.33) | T (lc) | B | N | **P** | A/T | Gca/Aca | 62 | 8 |
| C13712T | _ | 1 (3.33) | T (lc) | B | N | N | A/V | gCc/gTc | -78 | 1 |
| C13760T | 1 (3.33) | _ | T (lc) | B | N | N | A/V | gCa/gTa | 27 | 1 |
| G13889A | 1 (3.33) | _ | T (lc) | B | N | N | C/Y | tGc/tAc | -50 | 2 |
| A13966G | _ | 2 (6.66) | T (lc) | B | N | N | T/A | Acg/Gcg | -49 | 1 |
| T14000A | 1 (3.33) | _ | T (lc) | **PRD** | **D** | N | L/Q | cTa/cAa | -66 | 4 |
| A14128G | _ | 3 (10.00) | T (lc) | **PSD** | N | N | T/A | Acc/Gcc | -40 | 1 |
| *MT-ND6* of 60 matched pairs | | | | | | | | |  | |
| A14312G | 1 (1.66) | _ | T | B | N | N | V/A | gTa/gCa | -16 | 1 |
| T14502C | 1 (1.66) | 1 (1.66) | B | N | N | N | I/V | Att/Gtt | -1 | 7 |
| C14553T | 3 (5.00) | 1 (1.66) | B | N | N | N | V/I | Gtt/Att | -64 | 6 |
| *MY-CYB* of 60 matched pairs | | | | | | | | |  | |
| C14766T | 57 (95.00) | 59 (98.33) | D (lc) | B | **D** | N | T/I | aCt/aTt | 50 | 5 |
| G14861A | 1 (1.66) | _ | T (lc) | B | **D** | N | A/T | GCc/ACc | 19 | 1 |
| A14927G | _ | 1 (1.66) | D (lc) | B | **D** | N | T/A | Acc/Gcc | -62 | 1 |
| G15110A | 2 (3.33) | 3 (5.00) | T (lc) | B | N | N | A/T | Gca/Aca | 55 | 1 |
| G15119A | 1 (1.66) | _ | D (lc) | B | **D** | **P** | A/T | Gca/Aca | 34 | 6 |
| G15314A | 3 (5.00) | 4 (6.66) | T (lc) | B | **D** | **P** | A/T | Gca/Aca | 14 | 1 |
| A15326G | 56 (93.33) | 59 (98.33) | T (lc) | B | N | N | T/A | Aca/Gca | 55 | 1 |
| G15431A | 6 (10.00) | 5 (8.33) | D (lc) | B | **D** | **P** | A/T | Gcc/Acc | 62 | 1 |
| C15452A | 1 (1.66) | 2 (3.33) | B | N | N | N | L/I | Ctt/Att | -12 | 1 |
| G15497A | 1 (1.66) | _ | N | B | **D** | N | G/S | Ggc/Agc | 81 | 4 |
| G15803A | 1 (1.66) | _ | T (lc) | B | N | **P** | V/M | Gta/Ata | 18 | 2 |
| G15884A | 3 (5.00) | 4 (6.66) | T (lc) | **PSD** | **D** | **P** | A/T | Gcc/Acc | -79 | _ |

Variants identified as deleterious/damaging by SIFT and PolyPhen 2, and pathogenicity predicted by SIFT and PolyPhen 2/ reported by CADD and APOGEE are indicated in bold.

B: Benign, D: deleterious, lc: low confidence, N: neutral; P: pathogenic; PRD: probabaly damaging, PSD: possibly damaging, T: tolerated, U: unknown
