# Supplementary material for: Mitochondrial genome in sporadic breast cancer: A case control study and a proteomic analysis in a Sinhalese cohort from Sri Lanka
Source: PLoS One. 2023 Feb 9;18(2):e0281620. doi: 10.1371/journal.pone.0281620 (PMC9910733; doi:10.1371/journal.pone.0281620)
Supplement: S6 Table — (DOCX) [file pone.0281620.s008.docx]

**Supplementary Table 6: Shared haplotypes**

| Haplotype | Variants | Patients n | Controls n | |
| --- | --- | --- | --- | --- |
| HI. | A73G, C150T, A263G, T489C, C511T, A750G, A1438G, G1664A, A2706G, T3336C, A4769G, A916G, A6040G, C7028T, G8251A, A8701G, A8739G, A8860G, T9540C, A10398G, C10400T, T10873C, G11719A, G12007A, C12705T, A13651G, T14290C, C14766T, T14783C, G15043A, G15301A, A15326G, A15924G, C16223T, A16289, T16519 | 1 | 2 | |
| HII. | A73G, C150T, A263G, 310insC , T489C, C511T, A750G, A1438G, G1664A, A2706G, T3336C, A4769G, A4916G, A6040G, C7028T, G8251A, A8701G, A8739G, A8860G, T9540C, A10398G, C10400T, T10873C, G11719A, G12007A, C12705T, A13651G, T14290C, C14766T, T14783C, G15043A, G15301A, A15326G, A15924G, C16223T, A16289G, T16519C | 1 | 1 |  |
| HIII. | A73G, T199C, A263G, 310insC , T482C, T489C, A750G, A1438G, A2706G, A4769G, T5426C, A5432G, C7028T, A8701G, A8860G, T9540C, A10398G, C10400T, C10670T, T10873C, G11719A, G12561A, C12705T, C14766T, T14783C, G15043A, G15301A, A15326G, A15924G, T16093C, C16223T, C16320T, T16519C | 1 | 1 |  |
