# Supplementary material for: Mitochondrial genome in sporadic breast cancer: A case control study and a proteomic analysis in a Sinhalese cohort from Sri Lanka
Source: PLoS One. 2023 Feb 9;18(2):e0281620. doi: 10.1371/journal.pone.0281620 (PMC9910733; doi:10.1371/journal.pone.0281620)
Supplement: S7 Table — (DOCX) [file pone.0281620.s009.docx]

**Supplementary Table 7: Detail description of the mt haplogroups in 30 matched pairs analysed using next generation sequencing**

|  | Haplogroup, (% total population) | Sub-clade | Patients, n | Controls, n |
| --- | --- | --- | --- | --- |
|  |  |  |  |  |
| M |  | M2b | 1 |  |
|  |  | M3c1a | 1 |  |
|  |  | M4a | 2 |  |
|  |  | M4'67 |  | 1 |
|  |  | M5a |  | 1 |
|  |  | M6a |  | 1 |
|  |  | M6a1a |  | 1 |
|  |  | M6a1b |  | 1 |
|  |  | M18'38 | 1 |  |
|  |  | M30 | 3 | 1 |
|  |  | M30c | 1 | 1 |
|  |  | M30+16234 | 1 |  |
|  |  | M35a1 | 2 | 3 |
|  |  | M36 |  | 1 |
|  |  | M36a | 1 |  |
|  |  | M37e |  | 1 |
|  |  | M38a |  | 1 |
|  |  | M40 |  | 1 |
|  |  | M65a+@16311 | 4 | 4 |
|  | M (58.33%) | Total | 17 | 18 |
| N | N (1.66%) | N5a | 1 |  |
|  |  | U2a1a |  | 1 |
|  |  | U2b2 | 1 | 2 |
|  |  | U2c1 | 1 | 1 |
|  |  | U2c1a | 1 |  |
|  |  | U2e1a1 |  | 1 |
|  |  | U3b1a1 |  | 1 |
|  |  | U3b3 |  | 1 |
|  |  | U1a1c1d | 2 |  |
|  |  | U7 |  | 1 |
|  |  | U7a3a | 1 | 1 |
|  | U (26.66%) | U7a2 | 1 |  |
|  |  | R30b2a | 1 |  |
|  |  | R31b |  | 1 |
|  |  | R8a1+16093 | 1 |  |
|  | R (8.33%) | R6a | 1 | 1 |
|  | HV (1.66%) | HV12b1 | 1 |  |
|  | H (3.33%) | H6a1a | 1 | 1 |
|  |  | Total | 13 | 12 |
|  |  |  |  |  |
| Total |  |  | 30 | 30 |
