# Supplementary material for: Mitochondrial genome in sporadic breast cancer: A case control study and a proteomic analysis in a Sinhalese cohort from Sri Lanka
Source: PLoS One. 2023 Feb 9;18(2):e0281620. doi: 10.1371/journal.pone.0281620 (PMC9910733; doi:10.1371/journal.pone.0281620)
Supplement: S8 Table — (DOCX) [file pone.0281620.s010.docx]

**Supplementary Table 8: List of Acronyms**

Asn - Asparagine

ATP - Adenosine triphosphate

BAM - Binary alignment map

BQSR - Base quality score recalibration

*BRCA1 -* Breast Cancer gene 1

*BRCA2 -* Breast Cancer gene 2

BWA-MEM - Burrows-Wheeler Aligner - Maximal Exact Match

DNA - deoxyribonucleic acid

GATK - Genome Analysis Tool Kit

H-bonds - Hydrogen bonds

IGV - Integrative Genomics Viewer

MAF - Mutant allele fraction

MD - Molecular dynamics

mt - Mitochondrial

mtDNA - Mitochondrial DNA

*MT-ATP6 -* Mitochondrially encoded ATP synthase membrane subunit 6

*MT-ATP8 -* Mitochondrially Encoded ATP Synthase Membrane Subunit 8

*MT- CO1 -* Mitochondrially Encoded Cytochrome C Oxidase I

*MT-CO2 -* Mitochondrially Encoded Cytochrome C Oxidase II

*MT-CO3 -* Mitochondrially Encoded Cytochrome C Oxidase III

*MT-CYB -* Mitochondrially Encoded Cytochrome B

*MT-ND1 -* Mitochondrially Encoded NADH:Ubiquinone Oxidoreductase Core Subunit 1

*MT-ND2 -* Mitochondrially Encoded NADH:Ubiquinone Oxidoreductase Core Subunit 2

*MT-ND3 -* Mitochondrially Encoded NADH:Ubiquinone Oxidoreductase Core Subunit 3

*MT-ND4 -*  Mitochondrially Encoded NADH:Ubiquinone Oxidoreductase Core Subunit 4

*MT-ND4L -* Mitochondrially Encoded NADH:Ubiquinone Oxidoreductase Core Subunit 4L

*MT-ND5 -* Mitochondrially Encoded NADH:Ubiquinone Oxidoreductase Core Subunit 5

*MT*-*ND6 -* Mitochondrially Encoded NADH:Ubiquinone Oxidoreductase Core Subunit 6

*MT-RNR1 -* Mitochondrially Encoded 12S Ribosomal Ribonucleic acid

*MT-RNR2 -* Mitochondrially Encoded 16S Ribosomal Ribonucleic acid

*MT-TD -* Mitochondrially encoded Transfer Ribonucleic acid aspartic acid

*MT-tRNA –* Mitochondrial Transfer Ribonucleic acid

*MT-TT -* Mitochondrially encoded Transfer Ribonucleic acid threonine

MT-TL2 - Mitochondrially encoded Transfer Ribonucleic acid leucine 2

NGS - Next generation sequencing

NOS - Not otherwise specified

NST - No special type

PC - Principal component

PCA - Principal component analysis

POPE - 1-Palmitoyl-2-oleoyl-sn-glycero-3-phosphoethanolamine

PCR - Polymerase Chain Reaction

rCRS - revised Cambridge Reference Sequence

RMSD - The root means square deviation

Rg - Radius of gyration

SAM - Sequence alignment mapping

SD - Standard deviation

SNP - Single nucleotide polymorphism

TMH - Transmembrane helices

RMSF - Root mean square fluctuation

VEP - Variant Effect Predictor
